# Evaluation of Factors Affecting Size and Size Distribution of Chitosan-Electrosprayed Nanoparticles

**Published:** 2017

**Authors:** Morteza Abyadeh, Ali Akbar Karimi Zarchi, Mohammad Ali Faramarzi, Amir Amani

**Affiliations:** 1. Department of Medical Nanotechnology, Faculty of Advanced Technologies in Medicine, Tehran University of Medical Sciences, Tehran, Iran; 2. Department of Pharmaceutical Biotechnology, Faculty of Pharmacy, Biotechnology Research Center, Tehran University of Medical Sciences, Tehran, Iran; 3. Medical Biomaterials Research Center, Tehran University of Medical Sciences, Tehran, Iran

**Keywords:** Chitosan, Electrospray, Experimental design Nanoparticles, Particle size

## Abstract

**Background:**

Size and size distribution of polymeric nanoparticles have important effect on their properties for pharmaceutical application. In this study, Chitosan nanoparticles were prepared by electrospray method (electrohydrodynamic atomization) and parameters that simultaneously affect size and/or size distribution of chitosan nanoparticles were optimized.

**Methods:**

Effect of formulation/processing three independent formulation/processing parameters, namely concentration, flow rate and applied voltage was investigated on particle size and size distribution of generated nanoparticles using a Box–Behnken experimental design.

**Results:**

All the studied factors showed important effects on average size and size distribution of nanoparticles. A decrease in size and size distribution was obtainable with decreasing flow rate and concentration and increasing applied voltage. Eventually, a sample with minimum size and polydispersity was obtained with polymer concentration, flow rate and applied voltage values of 0.5 %w/v, 0.05 *ml/h*r and 15 *kV*, respectively. The experimentally prepared nanoparticles, expected having lowest size and size distribution values had a size of 105 *nm*, size distribution of 36 and Zeta potential of 59.3 *mV*.

**Conclusion:**

Results showed that optimum condition for production of chitosan nanoparticles with the minimum size and narrow size distribution was a minimum value for flow rate and highest value for applied voltage along with an optimum chitosan concentration.

## Introduction

Biodegradable polymeric nanoparticles (NPs) have been used widely as drug carriers, especially for oral and pulmonary drug delivery purposes ^1^. Because of their small size, they can solubilize a concentrated payload of therapeutic agent, improve drug stability and bioavailability, and provide sustained delivery. Moreover, use of biodegradable and biocompatible materials decreases the risk of unwanted toxicities and adverse effects ^2^.

Natural polymers have been widely used as drug carrier in the literature. Chitosan (CS) is a natural polymer with properties such as biodegradability, biocompatibility, low toxicity ^3^, efficacious delivery of therapeutic agent ^4^, mucoadhesivity ^5^ and ability to facilitate the macromolecules permeation through the epithelia by opening tight junctions ^6^. Chitosan nanoparticles (NPs) are being extensively investigated for delivery of drugs, proteins/peptides and genes ^7–9^.

Recently, fabrication of polymeric NPs using electrospraying (electrohydrodynamic atomization) has received a great deal of attention for pharmaceutical purposes. Electrospray is based on the ability of electric field to create electrostatic forces within a liquid droplet to deform the droplet by repulsion between the coions. This results in an outwardly directed force when a charge is induced on the surface of the liquid ^10^. Fabrication of polymeric particles by electrospray has the potential to overcome limitations of other techniques to provide reproducibly loaded nano- and microparticles ^11–14^. Compared with approaches such as solvent evaporation and emulsification, electrospray has the advantages of narrower particle size distribution, higher drug-loading efficiency and lack of solvent residue ^15,16^. Additionally, this cost effective, simple and one-step technique does not require the use of template or surfactant and employs mild condition for sensitive therapeutic agents ^17,18^.

Size and size distribution of generated nanoparticles in electrospray method may be affected by many variables. By optimizing solution, process and environmental parameters, one may obtain a desired size and size distribution. No need to mention that size and size distribution of polymeric nanoparticles notably influence properties such as blood circulation time, bioavailability and cellular uptake ^19–21^. Few researches have studied parameters that affect size and/or size distribution of nanoparticles so far which are produced by electrospray. Size and size distribution of nanoparticles is greatly influenced by solution properties including polymer molecular weight ^22^, surface tension, conductivity, polymer concentration and acid concentration ^1,23^ as well as process parameters such as flow rate, needle gauge, applied voltage and distance between the electrodes ^24^. For instance, Enayati *et al* reported that lowest PLGA particle size and size distribution was obtained at concentration of 5 (%wt) in range of study (2% wt-10% wt) ^25^. In a study by our group, mean particle diameter decreased as both flow rate and polymer concentration were reduced ^26^. However, majority of such studies are PLGA-based reports. Very limited reports on chitosan are found which include chitosan/ampicilin (520 *nm*) ^27^, doxorubicin/chitosan/tripoly-phosphate (200 *nm*) ^28^, chitosan/indomethacin (340 *nm*) ^22^ as well as chitosan alone, (167 *nm*) ^23^ and (124 *nm*) ^1^. However, the result of investigation of parameters that simultaneously affect size and/or size distribution of chitosan nanoparticles produced with electrospray process has not been reported yet. More importantly, there is no detailed report focusing on size distribution of polymeric nanoparticles with this method.

Our investigation focused on the concurrent effect of three variables including polymer concentration, applied voltage and flow rate on nanoparticles size and size distribution to obtain the optimum conditions [(*i.e*. smaller size and narrowed Size Distribution (SD)].

## Materials and Methods

### Materials

High molecular weight chitosan (CS) (MW=500 *kDa*, DD=85%) was purchased from Zhengzhou Sigma Chemical Co. (China). Acetic acid was purchased from Merck Chemicals (Germany).

### Solution properties

17 solutions of chitosan in aqueous acetic acid were prepared with a different chitosan concentration. Acetic acid concentration was fixed at 50% (*v/v*) in all samples.

### Preparation of chitosan nanoparticles

To prepare chitosan nanoparticles, solutions with concentrations of 0.1, 0.4 and 0.7 (%w/v) were prepared by dissolving chitosan polymer into aqueous acetic acid solvent. The polymer solutions were stirred magnetically for 1 *hr* at room temperature before electrospraying. Then, the polymer solutions were transferred into a 2 *ml* plastic syringe and continuously forced through the spraying nozzle which was wired to the high voltage power supply (13, 14 and 15 *kV*) using a programmed pump (flow rate: 0.05, 0.2 and 0.35 *ml/hr*). For all samples, the needle gauge and the distance between nozzle and collector were kept at 27 *g* and 10 *cm*, respectively. Droplets were formed at the nozzle tip, in form of a cone called Taylor cone, when the electrical field overcame the surface tension of the polymer solution. The solvent was evaporated before reaching the collector to deposit the polymer in form of nanoparticles on the collector (*i.e*. alumina foil).

### Particle size and zeta potential

Size and morphology of nanoparticles were determined by scanning electron microscopy (SEM) (ZEISS DSM 960A Oberkochen, Germany). Samples were sputter coated with gold (20 *kV* for 3 *min*). Zeta potential of optimized nanoparticle was measured using a Zetasizer (Nano-ZS, Malvern Instruments Ltd., UK).

### Experimental design

Determination of the most important parameter and optimum level of each parameter with trial-and-error experiments is a time-consuming process. Therefore, Box-Behnken was employed as a response surface methodology (RSM) to optimize the three independent parameter levels. RSM is a statistical method for fitting the experimental data to a model for optimization ^29^.

Design-Expert (version 7.0.0, Stat-Ease, USA) was used to define the values of three independent parameters including applied voltage, chitosan concentration and flow rate in three levels as low (−1), basal (0) and high (+1), as given in table 1. Total number of experiments was 17, including 12 factorial points and 5 replicates at the center point for estimation of pure error sum of squares (Table 2).

**Table 1.** Variables used in Box–Behnken design

**Independent variable**	**Levels**		
	−1	0	1
**Applied voltage (*kV*)**	13	14	15
**Flow rate (*ml/hr*)**	0.05	0. 2	0.35
**Concentration (%w/v)**	0.1	0.4	0.7
**Dependent variables**	Constraints		
**Y_1_= particle size (*nm*)**	Minimize		
**Y_2_ = size distribution**	Minimize		

**Table 2.** Box–Behnken experimental design in 17 runs and the correspondent responses

**Run no.**	**Independent variables**			**Dependent variables**		
	**A**	**B**	**C**	**Y_1_ (size)**	**Y_2_ (size distribution)**
**1**	0	0	0	122	57
**2**	1	−1	0	122	53
**3**	1	1	0	171	68
**4**	0	0	0	119	48
**5**	0	0	0	121	51
**6**	−1	−1	0	121	43
**7**	0	0	0	122	67
**8**	0	−1	1	105	36
**9**	0	1	−1	170	78
**10**	1	0	1	131	53
**11**	−1	1	0	132	56
**12**	1	1	−1	170	84
**13**	−1	0	−1	137	59
**14**	0	−1	−1	139	63
**15**	0	1	1	142	71
**16**	−1	0	1	128	51
**17**	0	0	0	125	50

A) Concentration; B) Flow rate; C) Voltage.

Mathematical relationship of the response (Yi, particle size and size distribution) with the independent variables (Xi, concentration, applied voltage and flow rate) can be modeled by a second-order polynomial function model:

(1) $$\text{Y}=\beta_{0}+\beta_{1}{\text{X}}_{1}+\beta_{2}{\text{X}}_{2}+\beta_{3}{\text{X}}_{3}+\beta_{11}{\text{X}}_{1}^{2}+\beta_{22}{\text{X}}_{2}^{2}+\beta_{33}{\text{X}}_{3}^{2}+\beta_{12}{\text{X}}_{1}{\text{X}}_{2}+\beta_{13}{\text{X}}_{1}{\text{X}}_{3}+\beta_{23}{\text{X}}_{2}{\text{X}}_{3}$$

where Y is the predicted response, *β*_0_, intercept, *β*_1_, *β*_2_ and *β*_3_, linear coefficients, *β*_11_, *β*_22_ and *β*_33_, squared coefficients and *β*_12_, *β*_13_ and *β*_23_, the interaction coefficients of the equation and X_1_, X_2_ and X_3_ are the independent variables.

Contour plots and 3D graphs were used to show the relationship and interaction of independent variables with the dependent response. Software-proposed optimized samples to prepare nanoparticles, considering minimum value for particle size and size distribution were used to experimentally prepare the samples. The results were then compared with the predicted values by the software to investigate the ability of the model to estimate the optimum conditions.

## Results

Compared with conventional methods of generating aerosol droplets, electrospray is a straightforward technique which commonly produces less hetero-dispersed nanoparticles. In this approach, an electrical potential is applied on the nozzle. Above a certain critical charge, the electrical force overcame the liquid surface tension and broke the solution down to small electrically charged droplets that repel each other and produce a shower of fine polymer droplets ^30^. Nanoparticles with nearly monodispersed size distribution and spherical shapes can be produced by controlling the electrospraying parameters ^2^.

Different jet types may be obtained in an electrospary process; dripping, spindle, ramified-meniscus modes in low applied voltage and single cone-jet at an optimum voltage value as well as multi-jet at higher values are commonly observed. A stable single cone-jet mode is essential for production of nearly monodis-persed micro- and nanoparticles ^31^.

Our experiments were performed at atmospheric pressure and room temperature. The simultaneous effect of three variable parameters on nanoparticles size and size distribution was investigated. Chitosan nanoparticles were prepared with size and size distribution ranging from 105 to 171 *nm* and 27 to 84 (Table 2), respectively. To obtain the best lack of fit and model F-values, quadratic second-order polynomial equation was used.

The equations fitted to the data were as follows:

(2) $${\text{Y}}_{1}=+2386.92+263.00{\text{X}}_{1}-226.22{\text{X}}_{2}-316.55{\text{X}}_{3}+211.11{\text{X}}_{1}{\text{X}}_{2}-25.00{\text{X}}_{1}{\text{X}}_{3}+10.00{\text{X}}_{2}{\text{X}}_{3}+95.56{{\text{X}}_{1}}^{2}+271.11{{\text{X}}_{2}}^{2}+11.10{{\text{X}}_{3}}^{2}$$

(3) $${\text{Y}}_{2}=+1430.08+288.75{\text{X}}_{1}-401.67{\text{X}}_{2}-191.29{\text{X}}_{3}-19.17{\text{X}}_{1}{\text{X}}_{3}+33.33{\text{X}}_{2}{\text{X}}_{3}+6.54{{\text{X}}_{3}}^{2}$$

where Y_1_ and Y_2_ code size and size distribution and X_1_, X_2_ and X_3_ are concentration, flow rate and voltage, respectively. The coefficients of determination (R^2^) of the model for size and size distribution were 0.99 and 0.88, respectively, with adjusted R^2^ of 0.98 and 0.81, respectively, implying capability of the model to predict the two responses.

**Figure 1.**
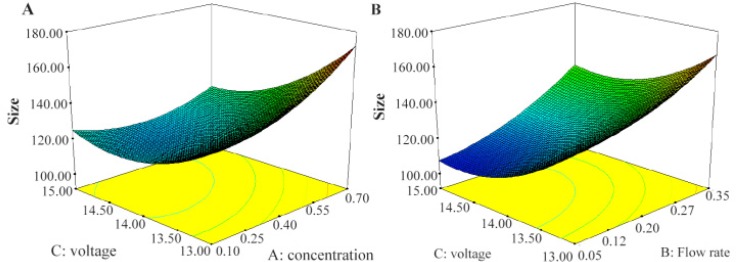
Effects of voltage and concentration; A) as well as voltage and flow rate; B) on size of nanoparticles.

**Figure 2.**
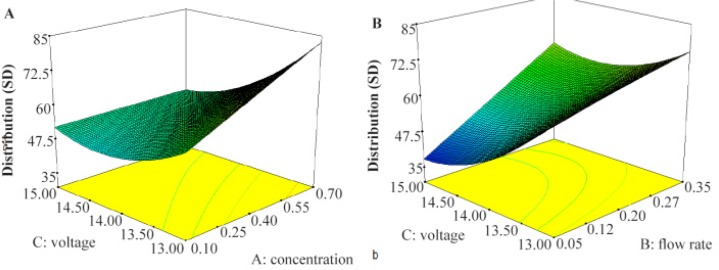
Effects of voltage and concentration: A) as well as voltage and flow rate; B) on nanoparticles size distribution.

The lack of fit F-values model for the size and size distribution were 0.20 and 0.95, respectively, indicating insignificance relative to the pure error and significant models, respectively ^32^. ANOVA results for two responses are shown in tables 3 and 4. Three-dimensional response surface plots, generated by the software which indicates the relation of the independent variables with the responses, are demonstrated in figures 1 and 2. In each plot, the interaction of two independent variables is investigated simultaneously while the third variable is at its middle-level value.

**Table 3.** ANOVA results for size as the responses

**Source**	**Sum of squares**	**df**	**Mean square**	**F value**	**p-value probe>F**
**Model**	5971.64	9	663.52	87.14	<0.0001
**A-Concentration**	722.00	1	722.00	94.82	significant
**B-Flow rate**	2048.00	1	2048.00	268.97	<0.0001
**C*-*Voltage**	1512.50	1	1512.50	198.64	<0.0001
**AB**	361.00	1	47.41	0.0002	<0.0001
**AC**	225.00	1	29.55	0.0010	
**BC**	9.00	1	1.18	0.3130	
**A^2^**	311.41	1	40.90	0.0004	
**B^2^**	156.67	1	20.58	0.0027	
**C^2^**	518.78	1	68.13	< 0.0001	
**Residual**	53.30	7	7.61		
**Lack of Fit**	34.50	3	11.50	2.45	0.2037
**Pure Error**	18.80	4	4.70		not significant
**Cor Total**	6024.94	16			

R-Squared 0.9912, Adj R-Squared 0.9798, Pred R-Squared 0.9035.

**Table 4.** ANOVA results for size distribution as the responses

**Source**	**Sum of squares**	**df**	**Mean square**	**F value**	**p-value probe>F**
**Model**	2140.24	6	356.71	12.75	0.0004
**A-Concentration**	300.12	1	300.12	10.72	significant
**B-Flow rate**	760.50	1	760.50	27.17	0.0084
**C*-*Voltage**	666.13	1	666.13	23.80	0.0004
**AC**	132.25	1	4.73	0.0548	0.0006
**BC**	100.00	1	3.57	0.0880	
**C^2^**	181.24	1	6.48	0.0291	
**Residual**	279.88	10	27.99		
**Lack of Fit**	68.68	6	11.45	0.22	0.9518
**Pure Error**	211.20	4	52.80		not significant
**Cor Total**	2420.12	16			

R-Squared 0.8844, Adj R-Squared 0.8150, Pred R-Squared 0.7527

Figure 1A shows the effect of applied voltage and concentration on the size of nanoparticles produced by electrospray. The findings show that particles size decreases with increase in applied voltage and decrease the concentration that is against the other report focusing on the role of applied voltage in spray mode and after forming stable cone jet, increase in applied voltage makes slight decrease in size of particles ^2,25^.

As shown, by increasing the concentration, the size increases. Literature shows that by manipulating chitosan concentration, some properties of the solution such as viscosity and conductivity ^1^ are influenced which in turn affects the droplets diameter. The relation is explained by an equation suggested by Hartman *et al* ^32^:

$$\text{d}=\alpha \left(\frac{\rho {ɛ}_{0}Q^{4}}{{\text{I}}^{2}}\right)1/6$$

$$I\propto {\left(\gamma \text{KQ}\right)}^{1/2}$$

where d is the droplet diameter, α is a constant, ρ is the solution density, ε_0_ is the permittivity of vacuum, Q is the liquid flow rate, I is the current, γ is the surface tension in ambient air, and K is the liquid conductivity. From the equation, viscosity and conductivity have contrary impacts on the size. For instance, Zhang *et al* reported that increase in concentration of chitosan solution, increased the nanoparticles size as the increase in viscosity was more effective than increase in conductivity of solution ^1^.

Looking more closely to the diagram, in our work, decrease in chitosan concentration from 0.7 to 0.4 (%w/w) shows substantial decrease in nanoparticles size but decrease from 0.4 to 0.1 (%w/w) made no further decrease and even small increase in nanoparticles size. It is already reported that when chitosan concentration is low, changing the polymer concentration makes substantially more variation in viscosity compared with conductivity ^1^. So, at such situations, the decrease in viscosity as a function of decrease in chitosan concentration is more than that of conductivity. Thus, decrease in size is expected. However, further decrease in viscosity makes the electrospray process unstable, which could be an explanation to the observed size increase ^33^.

The second most important parameter after solution parameters in electrospraying process is probably flow rate ^25^, which together with solution parameters can control polymer entanglements and Coulomb fission; consequently, affect particles size and size distribution ^2^. As shown in figure 1B, in our study, reduction in flow rate makes decrease in particles size, as documented previously ^34^. When flow rate increases, the droplet size increases which makes faster movement of droplets towards the collector. Thus, solvent may not evaporate completely when reaches the collector. This makes agglomeration of wet nanoparticles which in turn leads to formation of larger particles ^22,24^.

From figure 1B, increasing the voltage from 13 to 15 *kV* makes a decrease in the size. This was described above and is in agreement with the previous results ^24^.

Figure 2 details the effect of independent variables on the size distribution. From figure 2A, voltage shows a reverse effect of size distribution. It seems applied voltage has important effect on size distribution of nanoparticles. Forming the stable single cone-jet mode is essential for near monodispersed nanoparticles production and can be obtained with optimized applied voltage. As other researchers showed, after forming stable cone jet, more increase in applied voltage makes multi-jet ^24^ appear in an irregular shape ^27^ and leads to increase in size distribution.

Also, as other researchers reported, size distribution of nanoparticles increases with increasing polymer concentration ^26,27^. Increase in concentration makes an increase in viscosity and decrease in conductivity as described above, makes cone jet unstable and leads to increase in size distribution.

Figure 2B indicates that decrease in flow rate makes an important decrease in size distribution. As other researchers show, the jet mode also depends on flow rate and increased flow rate needs increase in applied voltage to make cone jet mode stable that is essential for monodisperse particles formation ^22^.

### Optimization

**Figure 3.**
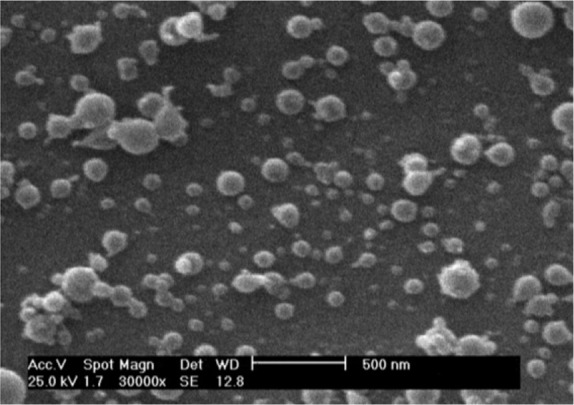
SEM image of chitosan nanoparticles

By solving equations 2 and 3 using the software, optimum values of 0.52 (%w/w), 0.05 (*ml/hr*) and 15 (*kV*) were given for chitosan concentration, flow rate and applied voltage, respectively. Using these values, 110 *nm* for size and 30 for size distribution were predicted by the software. Values were then used for experimental preparation of the samples. The results for three replicates were 110.6 (4.7) *nm*, 32 (5.8) and 59.3 *mV* for size, size distribution and zeta potential, respectively (Figure 3). These results show a good agreement between predicted value and obtained value.

## Discussion

Compared with conventional methods of generating aerosol droplets, electrospray is a straightforward technique which commonly produces less hetero-dispersed nanoparticles. In this approach, an electrical potential is applied on the nozzle. Above a certain critical charge the electrical force overcomes the liquid surface tension and breaks the solution down to small electrically charged droplets that repel each other and produce a shower of fine polymer droplets ^31^. Nanoparticles with nearly monodispersed size distribution and spherical shapes can be produced by controlling the electrospraying parameters ^2^.

Different jet types may be obtained in an electrospary process; dripping, spindle, ramified-meniscus modes in low applied voltage and single cone-jet at an optimum voltage value as well as multi-jet at higher values are commonly observed. A stable single cone-jet mode is essential for production of nearly mono-dispersed micro- and nanoparticles ^32^. Our experiments were performed at atmospheric pressure and room temperature. Reviewing the graphs indicate that decreased size and narrow size distribution could be obtained with decreasing flow rate and concentration and increasing applied voltage.

As shown in figure 1A and figure 1B by increase applied voltage and decrease the concentration and flow rate, size decrease, that is against other report that most role of applied voltage is on spray mode and after forming stable cone jet increase in applied voltage make slight decrease in size of particles ^2,24^.

Literature shows that by manipulating chitosan concentration, some properties of the solution such as viscosity and conductivity ^1^ is influenced which in turn affects the droplets diameter. The relation is explained by an equation suggested by Hartman *et al* ^32^:

$$\text{d}=\alpha \left(\frac{\rho {ɛ}_{0}Q^{4}}{{\text{I}}^{2}}\right)1/6$$

$$I\propto {\left(\gamma \text{KQ}\right)}^{1/2}$$

where d is the droplet diameter, α is a constant, ρ is the solution density, ε_0_ is the permittivity of vacuum, Q is the liquid flow rate, I is the current, γ is the surface tension in ambient air, and K is the liquid conductivity. From the equation, viscosity and conductivity have contrary impacts on the size. For instance, Zhang *et al* reported that increase in concentration of chitosan solution, increased the nanoparticles size as the increase in viscosity was more effective than increase in conductivity of solution ^1^.

Looking more closely to the diagram, in our work, decreases in chitosan concentration from 0.7 to 0.4 (%w/w) shows substantial decrease in nanoparticles size but decrease from 0.4 to 0.1 (%w/w) made no further decrease and even small increase in nanoparticles size. It is already reported that when chitosan concentration is low, changing the polymer concentration makes substantially more variation in viscosity compared with conductivity ^1^. So, at such situations, the decrease in viscosity as a function of decrease in chitosan concentration is more than that of conductivity. Thus, decrease in size is expected. However, further decrease in viscosity makes the electrospray process unstable, which could be an explain to the observed size increase ^33^.

The second most important parameter after solution parameters in electrospraying process is probably flow rate ^24^, which together with solution parameters can control polymer entanglements and Coulomb fission; consequently, affect particles size and size distribution ^2^. As shown in figure 1B, in our study reduction in flow rate makes decrease in particles size, as documented previously ^34^. When flow rate increases, the droplet size increases which makes faster movement of droplets towards the collector. Thus, solvent may not evaporate completely when reaches the collector. This makes agglomeration of wet nanoparticles which in turn leads to formation of larger particles ^22,24^.

As shown in figure 2A and 2B increase in applied voltage and decrease the concentration and flow rate makes decrease in size distribution.

Forming the stable single cone-jet mode is essential for near monodisperse nanoparticles production and can be obtained with optimize applied voltage. As other researcher showed after forming stable cone jet, more increase in applied voltage makes multi-jet ^24^, appear irregular shape ^27^ and leads to increase in size distribution. Also as other researcher reported size distribution of nanoparticles increase with increasing in polymer concentration ^26,27^. Increase in concentration make increase in viscosity and decrease in conductivity as above described, that make unstable cone jet and lead to increase in size distribution and as other researcher shows, the jet mode also depends on flow rate and increased flow rate needs increase in applied voltage to making stable cone jet mode that is essential for monodisperse particles formation ^22^.

## Conclusion

The aim of this study was to present optimized condition for production of solid chitosan nanoparticles using an electrospray device. The optimum condition for obtaining the minimum size and narrow size distribution was a minimum value for flow rate and highest value for applied voltage along with an optimum chitosan concentration. Afterwards, the optimum conditions were evaluated and solid chitosan nanoparticles were successfully prepared with size of 110.6 *nm*, size distribution of 32 and zeta potential of 59.3 by electrospray method which can be used for pharmaceutical applications.
